# Investigating the Association of Positive Affect and Rest-Activity Rhythm in Mid to Later Life Adults

**DOI:** 10.1093/geroni/igaf122.2744

**Published:** 2025-12-31

**Authors:** Meina Zhang, Jing Jiang, Kara Whitaker, Nai-Ching Chi, Sue Gardner, Boxiang Wang, Chooza Moon

**Affiliations:** UT Health, United States; University of Iowa, Iowa City, Iowa, United States; University of Iowa, Iowa City, Iowa, United States; University of Iowa, Iowa City, Iowa, United States; University of Iowa, Iowa City, Iowa, United States; University of Iowa, Iowa City, Iowa, United States; University of Iowa, Iowa City, Iowa, United States

## Abstract

Positive affect, the experience of positive emotions, influences sleep and physical activity among midlife and older adults. However, the relationship between positive affect and rest-activity rhythm (RAR)—which specifically refers to the overall pattern of sleep and physical activity observed throughout a 24-hour period—has understudied in midlife and older adults. Further, it is unknown whether age, health, and income influence this relationship. To address this gap, we analyzed data from the Midlife in the United States Survey (N = 308, mean age (SD) = 54.80 (12.05)) to determine how positive affect correlates with key RAR parameters: mesor (the average activity level), amplitude (maximum activity level), acrophase (timing of maximum activity level) and R-square (a measure of rhythmicity). Participants wore the Mini Mitter Actiwatch®-64 for one week to assess RAR, and positive affect was measured using the Positive and Negative Affect Schedule questionnaire. We used interaction terms in multiple regression models to test the moderating roles of age, health, and income. After accounting for demographic factors and self-perceived health status, our results showed a significant positive association between higher positive affect and better RAR parameters, including mesor (β = 16.18, p = 0.03), amplitude (β = 18.46, p = 0.02), and R-square (β = 0.03, p = 0.01). Our findings also showed that higher income strengthened the positive relationship between positive affect and mesor. However, age and health did not appear to moderate these associations. These findings highlight the association between psychosocial factors like positive affect and RAR and suggest potential for longitudinal studies with diverse samples to explore causality.

